# Testosterone Replacement Therapy Is Associated With Increased Incidence Rate of Vertebral Fractures: A Matched Retrospective Analysis

**DOI:** 10.5435/JAAOSGlobal-D-24-00248

**Published:** 2025-01-02

**Authors:** Manjot Singh, Mohammad Daher, Bassel G. Diebo, Alan H. Daniels, Michel A. Arcand

**Affiliations:** From the Warren Alpert Medical School, Brown University, Providence, RI (Singh and Daher), and the Department of Orthopedics, Brown University, Providence, RI (Dr. Diebo, Dr. Daniels, and Dr. Arcand).

## Abstract

**Background:**

Whether testosterone replacement therapy (TRT) can mitigate the risk of vertebral fractures has not been well-studied.

**Methods:**

PearlDiver was queried to identify patients with and without the history of TRT. Groups were matched 1:1 by demographic variables and 2-year vertebral fracture incidence rate was compared. Multivariate logistic regression was done to identify independent predictors of vertebral fractures.

**Results:**

Among 77,491 matched patients, mean age was 54.7 ± 10.4 years, 74.3% were males, and mean Charlson Comorbidity Index was 0.17 ± 0.54. Testosterone replacement therapy patients had higher rates of vertebral fractures (0.31% vs 0.04%, *P* < 0.001), and these rates were observed to increase with age. Both men alone (0.36% vs 0.04%, *P* < 0.001) and women alone (0.16% vs 0.03%, *P* < 0.001) on TRT had higher rates of vertebral fractures. Multivariate analysis revealed that TRT (OR = 7.7, 95%CI = 5.1-11.7, *P* < 0.001), as well as chronic kidney disease (OR = 1.4, 95%CI = 1.1-2.0, *P* = 0.026), alcohol abuse (OR = 2.5, 95%CI = 1.8-3.5, *P* < 0.001), and diphosphonate use (OR = 2.2, 95%CI = 1.4-3.5, *P* < 0.001), increased vertebral fracture rates.

**Conclusions:**

Exogenous testosterone use was associated with increased 2-year incidence of vertebral fractures. Although a causal relationship could not be established, our findings highlight the need to use screening measures, such as dual-energy X-ray absorptiometry (DEXA) scan, to identify patients at risk of vertebral fractures.

Vertebral fractures are a common sequelae of osteoporosis, malignancy, and trauma.^1^ They frequently occur at the T12 or L1 vertebral level and are 2 to 2.5 times more prevalent in women, especially in postmenopausal women ages 80 years or older.^2^ With the aging population, the overall prevalence of vertebral fractures is only expected to increase.^3^ The primary risk factors of vertebral fractures include advanced age, reduced bone mineral density, glucocorticoid use, low body weight, early menopause, and presence of comorbidities, such as diabetes and tobacco use, although other factors may have also been correlated.^4-6^ Vertebral fractures can induce substantial back pain, functional limitations, and pain-related disability and can predispose patients to subsequent fractures and impaired quality of life.^7,8^

Previous studies have shown that low levels of serum sex hormones, including estradiol and testosterone, can reduce bone mineral density in the elderly and increase the risk of fragility fractures.^9,10^ Conversely, testosterone replacement therapy (TRT), often used to manage symptomatic hypogonadism, can inhibit bone resorption and increase bone mass, particularly at the lumbar spine level.^11-14^ However, whether TRT can mitigate the increased rate of fragility fractures, especially vertebral fractures, seen in those with low total testosterone levels has not been studied well.

Recently, a randomized-control trial of middle-aged and older men with hypogonadism found that fracture incidence was numerically higher among those on TRT compared with those who received placebo.^15^ However, this study did not account for other risk factors associated with fragility fractures and did not examine fracture incidence in women. As such, this study aims to investigate the incidence of vertebral fractures among men and women of various age groups on TRT.

## Methods

### Study Design

This study was a retrospective review of the publicly available, national, all-payer PearlDiver Mariner165 Database (PearlDiver Technologies Inc). The Mariner data set contains more than 165 million U.S patients covered under commercial insurance, Medicare, Medicaid, government insurance, and self-pay between 2010 and Q1 2022. Patient records were retrieved using procedural and diagnostic codes from the International Classification of Diseases Ninth Revision (ICD-9) for patient records before October 2015 and 10th (ICD-10) Revision for records after its implementation. Institutional review board approval was not required because the data set contains nonidentifiable patient reports.

### Study Population

All patients ages 35 to 75 years who filled a prescription for exogenous TRT for a minimum period of three consecutive months and had at least 2 years of follow-up data available were identified through PearlDiver. A randomly generated control group of patients ages 35 to 75 years who had no history of TRT prescription and had at least 2 years of follow-up data available were similarly identified. Patients with a history of rheumatologic and autoimmune musculoskeletal disease, mitochondrial disease, and cancer were excluded. From these cohorts, 150,000 patients were randomly selected for the TRT group and the control group.

### Data Extraction

Patient demographics, including age, sex, Charlson Comorbidity Index (CCI), tobacco use, and diabetes, were extracted. Vertebral fracture incidence over the 2-year follow-up period was identified using ICD-9 and ICD-10 diagnostic codes.

### Statistical Analysis

All included TRT and control patients were summarized by patient demographics before and after matching 1:1 by age, sex, CCI, tobacco use, and diabetes. The rate of vertebral fractures was calculated for the TRT and control groups, overall, stratified by sex (ie, male or female) and stratified by age group (ie, 35-45, 46-55, 56-65, and 66-75 years). Univariate comparisons were made using chi squared tests for categorical variables and Student *t* tests for continuous variables. Multivariate logistic regression controlling for hypogonadism, TRT for a period of three consecutive months, obesity, morbid obesity, chronic kidney disease, alcohol abuse, and osteoporosis was done to identify the independent predictors of vertebral fractures. All analyses were conducted using the built-in R 4.0.3 statistical software (R Foundation for Statistical Computing, https://www.R-project.org/) within PearlDiver, with *P* value of <0.05 indicating statistical significance.

## Results

### Patient Characteristics

In total,150,000 patients were included in each of the TRT and control groups. The mean age (55.2 ± 10.5 vs 56.1 ± 11.7, *P* < 0.001) and CCI (0.80 ± 1.44 vs 0.12 ± 0.46, *P* < 0.001), as well as the frequency of male sex (85.6% vs 42.8%, *P* < 0.001), reported tobacco users (33.1% vs 30.7%, *P* < 0.001), and patients with diabetes (40.4% vs 37.8%, *P* < 0.001), were higher in the TRT group. After matching, 77,491 patients were retained in each group, with a mean age of 54.7 ± 10.4 years, 74.3% being men, mean CCI was 0.17 ± 0.54, 34.5% reported as tobacco users, and 36.2% with a diagnosis of diabetes (Table 1).

**Table 1:** Demographic and Baseline Characteristics of the Matched and Nonmatched Cohorts

	Nonmatched			Matched		
	TRT	Control	*P*	TRT	Control	*P*
Count	150,000	150,000		77,491	77,491	
Age (yrs)	55.17 (10.5)	56.07 (11.69)	**<0.001**	54.68 (10.38)	54.68 (10.38)	1.000
CCI	0.80 (1.44)	0.12 (0.46)	**<0.001**	0.17 (0.54)	0.17 (0.54)	1.000
Male sex	128,457 (85.64)	64,118 (42.75)	**<0.001**	57,554 (74.27)	57,554 (74.27)	1.000
Tobacco use	49,695 (33.13)	46,090 (30.73)	**<0.001**	26,723 (34.49)	26,723 (34.49)	1.000
Diabetes	60,663 (40.44)	56,697 (37.80)	**<0.001**	28,026 (36.17)	28,026 (36.17)	1.000

Bold values indicate statistical significance at *p* < 0.05. TRT = testosterone replacement therapy, CCI = Charlson Comorbidity Index.

### Incidence of Vertebral Fractures

In general, TRT patients had higher rates of vertebral fractures than the control patients (0.31% vs 0.04%, *P* < 0.001). After stratifying by sex, both men alone (0.36% vs 0.04%, *P* < 0.001) and women alone (0.16% vs 0.03%, *P* < 0.001) on TRT had higher rates of vertebral fractures than the matched control patients. After stratifying by age, the incidence rate was observed to increase with age. Men of all age groups on TRT had higher rates of vertebral fractures than the matched control patients (*P* < 0.001). However, only women of age groups 46 to 55 years (0.14% vs 0.00%, *P* = 0.003) and 56 to 65 years (0.19% vs 0.03%, *P* = 0.005) on TRT had higher rates of vertebral fractures than the matched control patients, with no statistically significant difference in the remaining age categories (Table 2).

**Table 2:** Incidence of Vertebral Fractures at 2 Years

Variable	Count	TRT	Control	*P*
Total	77,491	237 (0.31)	31 (0.04)	**<0.001**
35-45 yrs	17,027	42 (0.25)	8 (0.05)	**<0.001**
46-55 yrs	23,728	69 (0.29)	10 (0.04)	**<0.001**
56-65 yrs	22,488	78 (0.35)	3 (0.01)	**<0.001**
66-75 yrs	14,248	48 (0.34)	10 (0.08)	**<0.001**
Males	57,554	206 (0.36)	25 (0.04)	**<0.001**
35-45 yrs	14,180	40 (0.28)	8 (0.06)	**<0.001**
46-55 yrs	16,575	59 (0.36)	10 (0.06)	**<0.001**
56-65 yrs	15,467	65 (0.42)	1 (0.01)	**<0.001**
66-75 yrs	11,332	42 (0.37)	6 (0.05)	**<0.001**
Females	19,937	31 (0.16)	6 (0.03)	**<0.001**
35-45 yrs	2847	2 (0.07)	0 (0.00)	0.480
46-55 yrs	7153	10 (0.14)	0 (0.00)	**0.003**
56-65 yrs	7021	13 (0.19)	2 (0.03)	**0.005**
66-75 yrs	2916	6 (0.21)	4 (0.14)	0.290

Bold values indicate statistical significance at *p* < 0.05. TRT = testosterone replacement therapy.

### Predictors of Vertebral Fractures

After accounting for other confounding variables, such as hypogonadism, obesity, morbid obesity, chronic kidney disease, alcohol abuse, osteoporosis, and diphosphonate use, TRT for three consecutive months markedly increased the likelihood of vertebral fracture within 2 years of TRT (OR = 7.7, 95% CI = 5.1-11.7, *P* < 0.001). Other independent predictors included chronic kidney disease (OR = 1.4, 95% CI = 1.1-2.0, *P* = 0.026), alcohol abuse (OR = 2.5, 95% CI = 1.8-3.5, *P* < 0.001), and diphosphonate use (OR = 2.2, 95% CI = 1.4-3.5, *P* < 0.001; Table 3).

**Table 3:** Logistic Regression Predicting the Risk of Vertebral Fractures

Variable	OR (95% CI)	*P*
Hypogonadism	1.14 (0.87-1.49)	0.346
TRT	7.70 (5.09-11.65)	**<0.001**
Obesity	1.02 (0.76-1.36)	0.787
Morbid obesity	1.35 (0.92-1.97)	0.121
Chronic kidney disease	1.44 (1.05-1.98)	**0.026**
Alcohol abuse	2.51 (1.79-3.52)	**<0.001**
Osteoporosis	1.54 (0.89-2.67)	0.123
Diphosphonate use	2.20 (1.38-3.50)	**<0.001**

Bold values indicate statistical significance at *p* < 0.05. TRT = testosterone replacement therapy

## Discussion

This retrospective review investigated the relationship between exogenous testosterone administration and subsequent vertebral fractures. After matching by other important risk factors, the 2-year incidence rate of vertebral fractures was found to be 7.6 times higher among patients on TRT than among control patients. Furthermore, after stratification by age and sex, the incidence rate was observed to increase with age and was, in general, 2.3 times higher among men on TRT than among women on TRT. These findings demonstrate an important correlation between testosterone use and vertebral fractures that may have clinical implications on testosterone prescription among the elderly population.

Androgens, such as testosterone and dihydrotestosterone, play a vital role on bone mineral density. Biochemical studies have shown that androgens directly promote proliferation and differentiation of osteoblasts.^16^ At the same time, they indirectly inhibit receptor activator of nuclear factor kappa beta ligand (RANKL) secretion by osteoblastic precursors, which is needed to stimulate proliferation of osteoclasts.^17^ Together, this promotes bone formation and inhibits bone resorption, thus increasing overall bone mineral density. High bone mineral density is subsequently protective against osteoporotic vertebral fractures.^18^ Thus, it would be expected that patients on TRT would have lower rates of vertebral fractures. However, similar to the findings reported by Snyder et al.,^15^ this study conversely found higher incidence rates of vertebral fractures among TRT users. Although the effect of exogenous testosterone on bone mineral density was not explored in this study, previous biochemical research suggests that patients on TRT, in comparison to control patients, likely experienced an increase in their bone mineral density. As such, alternate explanations for this increase in the incidence rate of vertebral fractures in patients on TRT are required. Testosterone therapy is frequently used as a performance-enhancing drug to assist with body building and limitations in sexual function.^19,20^ In addition, testosterone has previously been shown to increase risk-taking and aggressive behavior.^21-24^ Patients on TRT may, thus, be more frequently engaging in activities that place them at an increased risk of vertebral fractures. However, further research would be needed to confirm behavioral differences in testosterone users and nonusers.

Prior studies have shown that the prevalence of vertebral fractures increases with age and that women, especially those older than 60 years, have higher rates of vertebral fractures than men of the same age group.^2^ Because of the relative decline in hypothalamic regulation and testicular function with age, men generally note a decrease in their testosterone levels over time.^25^ However, because of the onset of menopause at around the age of 51 years, women note a substantial decline in their testosterone levels with age.^26^ The rapid decline in bone mineral density that accompanies menopause among women in their late 40s or early 50s could explain why women have higher rates of vertebral fractures after the age of 60 years.^27^ Although this study similarly noted increasing incidence of vertebral fractures with age, men on TRT were interestingly found to have proportionally higher rates of vertebral fractures than women on TRT. This could potentially be due to differences in baseline testosterone levels and exogenous testosterone dosage among men and women. Men have biologically higher baseline levels of testosterone and may thus require higher dosages of exogenous testosterone to prevent fragility fractures associated with low testosterone levels.^9^ However, the specific dosage needs to be carefully monitored because excessively high levels of total testosterone, especially above 500 ng/dL, could potentially disrupt bone metabolism and have an adverse effect on bone mineral density.^28^ Testosterone levels, as well as TRT utilization, should be further studied to understand the relative difference in the rates of vertebral fractures by sex.

Although a thorough review of the risk factors associated with vertebral fractures was not done here, multivariate modeling in this study revealed that diphosphonate use, chronic kidney disease, and alcohol use, in addition to TRT use, can independently increase the likelihood of vertebral fractures. Both chronic kidney disease and alcohol use have previously been associated with the development of osteoporosis, which can subsequently predispose patients to fragility fractures.^29,30^ Indeed, when diphosphonate use was excluded from the regression model, osteoporosis was markedly associated with vertebral fractures. Bisphosphonates are frequently prescribed to treat osteoporosis and are, therefore, protective against osteoporosis at standard dosages but can, interestingly, increase fracture incidence at higher frequency dosages.^31,32^ This was confirmed with the addition of diphosphonate use in the model. Besides the ones studied here, many other factors, including physical inactivity, low body weight, and calcium and/or vitamin D deficiency, have been similarly associated with decreased bone mineral density and increased the risk of vertebral fractures.^33^ Further research investigating these factors in association with TRT should be conducted to confirm the role on exogenous testosterone on the subsequent risk of vertebral fractures.

This study has several potential limitations. First, this study is retrospective in nature and cannot be used to make any causal inferences between TRT and vertebral fractures. Second, the reliability of the generated PearlDiver queries relies heavily on accurate ICD coding of vertebral fractures across the two groups. Third, as described previously, several risk factors have been associated with fragility factors and not all of them were incorporated in the analyses. Fourth, the specific dosing, route of administration, changes in dosing, and compliance with the prescription were not evaluated and could affect total testosterone levels across patients. Finally, the effect of testosterone on bone mineral density and, specifically, T scores was not assessed and could supplement the current findings.

## Conclusions

In this retrospective matched-cohort study, exogenous testosterone use was associated with increased 2-year incidence rate of vertebral fractures. In particular, men, especially middle-aged and elderly men, were observed to have the highest reported incidence rates. Although a causal relationship between testosterone use and vertebral fractures could not be established here, our findings highlight the need to use screening measures, such as DEXA scan, to identify patients who may be at risk of vertebral fractures within this population. Further research should be conducted to investigate the effect of testosterone on bone mineral density, as well as the mechanism of injury that subsequently led to the development of vertebral fractures.
